# An Unusual Presentation of Retinal Detachment and Conjunctivitis: A Case Report

**DOI:** 10.5811/cpcem.2020.7.48292

**Published:** 2020-07-30

**Authors:** Bailey Pierce, Scott M. Alter, Kyle Gerakopoulos, Jeniel Parmar

**Affiliations:** * Florida Atlantic University Charles E. Schmidt College of Medicine, Division of Emergency Medicine, Boca Raton, Florida; † Boca Raton Regional Hospital, Department of Emergency Medicine, Boca Raton, Florida

**Keywords:** Conjunctivitis, retinal detachment, foreign body, corneal abrasion

## Abstract

**Introduction:**

Vision loss is an ophthalmologic emergency with broad differential requiring prompt medical attention.

**Case Report:**

We describe a 55-year-old male presenting to the emergency department (ED) with unilateral, painless visual field deficit with ipsilateral conjunctivitis induced by a presumed foreign body. The patient described a foreign body sensation nine days prior to developing visual changes. In the ED, the patient was diagnosed with a retinal detachment using point-of-care ultrasonography, and emergent ophthalmologic consultation was obtained.

**Conclusion:**

Concurrent retinal detachment and conjunctivitis in a patient is extremely rare. Healthcare providers should be aware that foreign body-induced conjunctivitis could lead to retinal detachment.

## INTRODUCTION

A total of 11.9 million visits to emergency departments (ED) from 2006–2011 were for eye-related issues.^1^ Among these visits, acute vision loss is an opthalmologic emergency with a large differential. Retinal detachment (RD) is one cause of painless acute vision loss that affects one in 10,000 annually.^2^ Although RD is associated with a number of risk factors, RD following corneal abrasion or conjunctivitis is not well documented. We describe a case of a 55-year-old man who presented to the ED with acute retinal detachment following eye injury and subsequent symptoms of conjunctivitis. Physicians should be aware that minor eye injury and ocular inflammation may present with delayed RD.

## CASE REPORT

A 55-year-old man with no significant past medical history presented to the ED for evaluation of right eye conjunctival injection, irritation, and painless visual field loss over the lower half of his vision in the ipsilateral eye. The patient stated that nine days prior a foreign body may have penetrated his right eye, for which he did not seek medical attention at that time. In the affected eye, he subsequently developed erythema, edema, purulent crusty drainage, itching, and a foreign body sensation. On day eight after the initial eye injury, the patient developed sudden-onset painless vision loss over the lower aspect of the right visual field. The following day, he presented to the ED with these symptoms. The patient denied blurry vision, floaters, or any past ophthalmological history.

On physical exam of the right eye, the patient had minimal conjunctival injection. Visual field deficits were appreciated over the lower temporal and lower nasal sides of the right eye. All remaining visual fields and visual acuity were intact. Fluorescein staining and Wood’s lamp exam did not reveal any foreign body, with negative Seidel sign. Point-of-care ocular ultrasonography showed retinal detachment of the right eye. The case was discussed with an ophthalmologist, who came to the ED, evaluated the patient, and arranged for next day follow-up and outpatient retinal repair.

## DISCUSSION

Retinal detachment is the separation of the neurosensory retina from the retinal pigment epithelium and results in retinal ischemia with progressive photoreceptor degeneration. Rhegmatogenous RD, the most common type, is caused by breaks in the retina.^3^ Patients with RD will endorse a sudden loss of vision that begins peripherally. Permanent vision loss, even with surgical repair, is likely when detachment progresses across the fovea with central vision loss.^2^ Larger retinal breaks can progress to central vision loss over days while smaller breaks may progress over weeks to months.^4^ Rhegmatogenous RD has several risk factors, or detachment may be secondary to ocular trauma.^3^ Minor eye injuries causing corneal abrasion or conjunctivitis are not well-documented risk factors for any type of RD.

The patient we present had symptoms of conjunctivitis preceding his RD. Although our patient described classic signs and symptoms of conjunctivitis, other ocular conditions may present similarly. Our first diagnostic challenge was attempting to determine an accurate diagnosis for the patient’s initial symptoms to better understand the pathogenesis of his RD. We first considered whether these symptoms were secondary to his presumed eye injury, a corneal abrasion, or from an infectious etiology.

In our patient, the initial eye injury could have led to a retained foreign body causing subsequent conjunctivitis. A retained foreign body could have caused continued eye irritation and inflammation until it fell out, coinciding with improvement of his symptoms the night prior to presentation. It is also possible the foreign body caused a corneal abrasion with subsequent inflammation and foreign body sensation. Corneal abrasions typically heal within 24–48 hours, accounting for the negative Wood’s lamp exam at the time of presentation.^5^ Alternatively, these symptoms could have been caused by an infectious etiology. Bacterial conjunctivitis often presents with a unilateral conjunctival injection with increased discharge and purulence, symptoms that our patient endorsed. The pathogen was likely introduced by the foreign body itself or by the patient attempting to remove the presumed foreign body.

Although not likely, we considered other infectious etiologies for our patient’s initial symptoms that also present with conjunctival injection and have more documented associations with RD. Corneal abrasions can become secondarily infected and cause keratitis. Keratitis may similarly present with conjunctival injection, foreign body sensation, and discharge, but often presents with pain and corneal opacity, which were not appreciated on our patient’s exam. Keratitis may rarely progress to endophthalmitis, which typically develops following cataract surgery, ocular trauma, or hematogenous spread.^6^ However, our patient’s eye injury was minor, he denied cataract surgery, and endophthalmitis is not self-limited and would be apparent on exam. Another disease process that can present with conjunctival injection is anterior uveitis. This could then develop into a panuveitis with involvement of the retina, and later progress to RD. While uveitis may have an infectious etiology, it is more commonly associated with systemic disorders that would be revealed in the history.^7^ As symptoms of these conditions overlap, and because our patient did not seek medical attention for his prior symptoms, we were unable to confirm his initial diagnosis. However, given the lack of risk factors and self-limited nature of our patient’s symptoms, it was more likely to be continued irritation from a foreign body, corneal abrasion, or conjunctivitis.

**CPC-EM Capsule**

What do we already know about this clinical entity?

The well-described risk factors for retinal detachment include older age, myopia, ocular trauma, and previous eye surgery.

What makes this presentation of disease reportable?

Though common complaints, conjunctivitis and corneal abrasions have not been previously reported as precursors to retinal detachment.

What is the major learning point?

Delayed presentation of retinal detachment may occur after minor eye injury and ocular inflammation.

How might this improve emergency medicine practice?

Blindness due to retinal detachment may be prevented if physicians consider this devastating disorder in patients presenting with conjunctivitis or corneal abrasion.

Next, we considered whether our patient’s initial eye injury, the foreign body, or conjunctivitis played a role in the development of RD. Ocular injury is a well-documented cause of RD but usually follows significant ocular trauma, including open-globe injuries and blunt trauma severe enough to cause contusion. Although RD may occur at the time of injury, it may also be delayed. One study found that for both open- and closed-globe injuries, roughly half the participants had a delayed presentation to RD, ranging from four days to nine years.^8^ While it is possible our patient’s initial eye injury caused a delayed RD, his description of the injury, if an injury at all, did not seem severe enough to have caused RD. Conversely, minor ocular injuries that cause corneal abrasions are not well documented to cause RD. It is possible though that the initial eye injury caused an abrasion that healed with formation of fibrous bands that acted as a nidus for delayed tractional RD.

Alternatively, a retained intraocular foreign body (IOFB) itself may have caused a RD through continued inflammation, direct toxicity, or secondary infection. Retained IOFB usually occurs following penetrating open-globe trauma. Risk factors for subsequent RD include delayed IOFB removal and foreign body located in the posterior segment.^9^ While our patient did not have an open-globe injury, there are cases in which an occult IOFB after minimal or no reported trauma caused RD. In a case series of three, patients initially presented with uveitis and were found to have a secondary RD. Initial ultrasound did not reveal IOFB, but later was discovered one to three weeks after onset of symptoms during surgical exploration. Unlike our patient whose symptoms nearly resolved prior to presentation, all three cases had progressively worsened until treatment.^10^

It is also possible that development of conjunctivitis led to our patient’s RD. There has been little published in the literature regarding RD acutely following conjunctivitis. *Chlamydia trachomatis* can cause a self-limited hyper-purulent conjunctivitis and has been associated with the development of RD. One case report described a patient presenting with decreased visual acuity and RD with subretinal fluid and conjunctival scrapings positive for chlamydia. However, this patient did not present with conjunctivitis symptoms, and the RD associated with chlamydia usually occurs after repeated or persistent exposure with development of conjunctival scaring and Herbert’s pits.^11^ These findings were not visualized in our patient, and RD has not been reported to acutely follow chlamydial conjunctivitis.

Another case report describes a patient who had an episode of conjunctival injection, epiphora, and no pain. The patient’s symptoms worsened, and a serous RD was found on exam. After a thorough history and extensive lab testing, he was diagnosed with idiopathic orbital inflammatory syndrome (IOIS).^12^ While this report is similar to our patient’s, IOIS is a diagnosis of exclusion requiring an extensive workup that would not be completed in the ED. In our patient, IOIS was also less likely in the setting of more obvious risk factors.

Finally, it is possible our patient’s RD was coincidental and not related to either his eye injury or conjunctivitis. Rhegmatogenous RD is more common in the fourth through sixth decades of life, and risk factors include myopia, cataract surgery, previous RD in the contralateral eye, lattice degeneration, and some hereditary disorders.^3^ In non-traumatic RD, one study found that posterior vitreous detachment occurs prior to RD in 87.6% of cases. This typically presents with flashers and floaters one-half to three weeks prior to visual field loss.^13^ Our patient did not have many of the previously stated risk factors, and he denied flashers and floaters.

## CONCLUSION

Our case is unusual because RD does not usually develop after conjunctivitis-like symptoms. Healthcare providers should be vigilant during their assessment of patients with ocular complaints. We have described concomitant presentation of conjunctivitis and retinal detachment.
